# Entanglement distribution in multi-particle systems in terms of unified entropy

**DOI:** 10.1038/s41598-017-01286-2

**Published:** 2017-04-25

**Authors:** Yu Luo, Fu-Gang Zhang, Yongming Li

**Affiliations:** 1 College of Computer Science, Shaanxi Normal University, Xi’an, 710062 China; 2 School of Mathematics and Information Science, Shaanxi Normal University, Xi’an, 710062 China

## Abstract

We investigate the entanglement distribution in multi-particle systems in terms of unified (*q*, *s*)-entropy. We find that for any tripartite mixed state, the unified (*q*, *s*)-entropy entanglement of assistance follows a polygamy relation. This polygamy relation also holds in multi-particle systems. Furthermore, a generalized monogamy relation is provided for unified (*q*, *s*)-entropy entanglement in the multi-qubit system.

## Introduction

Quantum entanglement is an important resource in quantum information theory. Different from classical correlations, this restricted shareability of entanglement in multi-particle systems is known as monogamy property. The more entanglement shared between two parties implies the less entanglement shared with the rest of the system. Monogamy property plays a crucial role in quantum cryptography: which restricts the quantity of information captured by an eavesdropper about the secret key to be extracted^1–3^. Monogamy property has also been discussed in the device-independent quantum information processing^4^, condensed matter physics^5^ and black-hole physics^6, 7^.

The study of monogamy property has a long history. The first monogamy relation was found by Coffman *et al*., who considered a three-qubit system *ABC* ^8^, and showed that the amount of entanglement (which is quantified by the squared concurrence) between *A* and *B*, plus the amount of entanglement between *A* and *C*, cannot be greater than the amount of entanglement between *A* and the pair *BC*. Further, Osborne *et al*. proved the squared concurrence follows a general monogamy inequality for the *N*-qubit system^1^. Monogamy inequalities for different entanglement measures have been noted, such as concurrence^9–12^, entanglement of formation^13, 14^, negativity^15–19^, Rényi entropy entanglement^20, 21^, and Tsallis entropy entanglement^22–24^. For the other physical resources, such as discord and steering, the monogamy property of them has also been discussed^25–28^.

As dual to monogamy property, polygamy property in multi-particle systems has arised many interests by researchers^15, 19, 22, 29, 30^. Polygamy property was first provided by using the concurrence of assistance to quantify the distributed bipartite entanglement in multi-qubit systems^29, 30^. Polygamy property has also considered in many entanglement measures, such as Rényi entropy^20^, Tsallis entropy^22, 31^ and convex-roof extended negativity^19^.

Unified (*q*, *s*)-entropy is an important entropic measure, which can be used in many areas of quantum information theory. In this paper, we investigate the entanglement distribution in multi-particle systems in terms of unified (*q*, *s*)-entropy. We find that for any tripartite mixed state, the unified (*q*, *s*)-entropy entanglement of assistance follows a polygamy relation. This polygamy relation also holds in multi-particle systems. Furthermore, a generalized monogamy relation is provided for unified (*q*, *s*)-entropy entanglement in the multi-qubit system.

## Results

This paper is organized as follows. In the first subsection, we recall the definition of unified (*q*, *s*)-entropy and discuss the properties of unified (*q*, *s*)-entropy entanglement. In the second subsection, we give our main results. We summarize our results in the third subsection.

### Unified (*q*, *s*)-entropy entanglement and unified (*q*, *s*)-entropy entanglement of assistance

Given a quantum state *ρ* in the Hilbert space ${\mathcal H}$. The unified (*q*, *s*)-entropy is defined as^32^

1 $${S}_{q,s}(\rho )=\frac{1}{(1-q)s}[Tr{({\rho }^{q})}^{s}-1]$$

for any *q*, *s* ≥ 0 such that $q\ne 1$ and $s\ne 0$.

When *s* tends to 1, the unified (*q*, *s*)-entropy converges to Tsallis entropy *T*_*q*_(*ρ*)^33^

2 $$\mathop{\mathrm{lim}}\limits_{s\to 1}\,{S}_{q,s}(\rho )=\frac{1}{1-q}[Tr({\rho }^{q})-1].$$

When *s* tends to 0, the unified (*q*, *s*)-entropy converges to Rényi entropy *R*_*q*_(*ρ*)^34^

3 $$\mathop{\mathrm{lim}}\limits_{s\to 0}\,{S}_{q,s}(\rho )=\frac{1}{1-q}\,\mathrm{ln}\,Tr({\rho }^{q}).$$

When *q* tends to 1, the unified (*q*, *s*)-entropy converges to von Neumann entropy *S*(*ρ*)^35^

4 $$\mathop{\mathrm{lim}}\limits_{q\to 1}\,{S}_{q,s}(\rho )=-Tr\rho \,\mathrm{ln}\,\rho .$$

Because the limits exist in the case of *q* → 1 and *s* → 0, we will use *q* = 1 and *s* = 1 to represent the limits in this paper. Now, let’s consider the entanglement in terms of the unified (*q*, *s*)-entropy. For any pure state ${|\psi \rangle }_{AB}$ in the Hilbert space ${ {\mathcal H} }_{{\mathscr{A}}}\otimes { {\mathcal H} }_{ {\mathcal B} }$ (it’s does not matter for the sizes of subsystem *A* and *B*), the unified (*q*, *s*)-entropy entanglement is defined as^36^

5 $${E}_{q,s}({|\psi \rangle }_{AB})={S}_{q,s}({\rho }_{A})$$

for any *q*, *s* ≥ 0.

For a mixed state *ρ*_*AB*_, the unified (*q*, *s*)-entropy entanglement can be defined via the convex-roof extension

6 $${E}_{q,s}({\rho }_{AB})=\,{\rm{\min }}\sum _{i}{p}_{i}{E}_{q,s}({|{\psi }^{i}\rangle }_{AB}),$$

where the minimum is taken over all possible ensembles $\{{p}_{i},{|{\psi }^{i}\rangle }_{AB}\}$ of *ρ*_*AB*_ with ${\sum }_{i}{p}_{i}=1$ and *p*_*i*_ ≥ 0. It is straightforward to verify that ${E}_{q,s}({\rho }_{AB}\mathrm{)=0}$ if and only if ${\rho }_{AB}$ is a separable state for $q,s\ge 0$.

When $s$ tends to 1, the unified $(q,s)$-entropy entanglement becomes Tsallis entanglement^31^. When $s$ tends to 0, the unified $(q,s)$-entropy entanglement becomes Rényi entanglement^20^. Especially, The unified $(q,s)$-entropy entanglement becomes the entanglement of formation when $q$ tends to 1. The entanglement of formation is defined as^37, 38^

7 $${E}_{f}({\rho }_{AB})=\,{\rm{\min }}\sum _{i}{p}_{i}{E}_{f}({|{\psi }^{i}\rangle }_{AB}),$$

where ${E}_{f}(|{\psi }_{AB}^{i}\rangle )=-Tr{\rho }_{A}^{i}\,\mathrm{ln}\,{\rho }_{A}^{i}=-Tr{\rho }_{B}^{i}\,\mathrm{ln}\,{\rho }_{B}^{i}$ is the von Neumann entropy, the minimum is taken over all possible ensembles $\{{p}_{i},{|{\psi }^{i}\rangle }_{AB}\}$ of *ρ*_*AB*_ with ${\sum }_{i}{p}_{i}=1$ and *p*_*i*_ ≥ 0.

As a dual quantity to the unified (*q*, *s*)-entropy entanglement, the unified (*q*, *s*)-entropy entanglement of assistance ((*q*, *s*)-EOA) can be defined as

8 $${E}_{q,s}^{a}({\rho }_{AB})=\,{\rm{\max }}\sum _{i}{p}_{i}{E}_{q,s}({|{\psi }^{i}\rangle }_{AB}),$$

where the maximum is taken over all possible ensembles $\{{p}_{i},{|{\psi }^{i}\rangle }_{AB}\}$ of *ρ*_*AB*_ with ${\sum }_{i}{p}_{i}=1$ and *p*_*i*_ ≥ 0. To understand (*q*, *s*)-EOA better, consider a tripartite pure state ${|\psi \rangle }_{ABC}$ shared among three parties referred to as Alice, Bob, and Charlie^39^. The entanglement supplier, Charlie, performs a measurement on his share of the tripartite state, which yields a known bipartite entangled state for Alice and Bob. Tracing over Charlie’s system yields the bipartite mixed state ${\rho }_{AB}=T{r}_{C}({|\psi \rangle }_{ABC}\langle \psi |)$ shared by Alice and Bob. Charlie’s aim is to maximize entanglement for Alice and Bob, and the maximum average entanglement he can create is the (*q*, *s*)-EOA.

### Concurrence and concurrence of assistance

For any pure state ${|\psi \rangle }_{AB}$ in the Hilbert space ${ {\mathcal H} }_{{\mathscr{A}}}\otimes { {\mathcal H} }_{ {\mathcal B} }$, the concurrence is defined as^40^

9 $${\mathscr{C}}({\rho }_{AB})=\sqrt{2(1-Tr{\rho }_{A}^{2})},$$

where $${\rho }_{A}=T{r}_{B}({\rho }_{AB})$$.

For a mixed state *ρ*_*AB*_, the concurrence can be defined via the convex-roof extension

10 $${\mathscr{C}}({\rho }_{AB})=\,{\rm{\min }}\sum _{i}{p}_{i}{\mathscr{C}}({|{\psi }^{i}\rangle }_{AB}),$$

where the minimum is taken over all possible ensembles $\{{p}_{i},{|{\psi }^{i}\rangle }_{AB}\}$ of *ρ*_*AB*_ with ${\sum }_{i}{p}_{i}=1$ and *p*_*i*_ ≥ 0.

As a dual quantity to concurrence, the concurrence of assistance (COA) can be defined as

11 $${{\mathscr{C}}}^{a}({\rho }_{AB})=\,{\rm{\max }}\sum _{i}{p}_{i}{\mathscr{C}}({|{\psi }^{i}\rangle }_{AB}),$$

where the maximum is taken over all possible ensembles $\{{p}_{i},{|{\psi }^{i}\rangle }_{AB}\}$ of *ρ*_*AB*_ with ${\sum }_{i}{p}_{i}=1$ and *p*_*i*_ ≥ 0.

### Analytical formula for two-qubit states

For a two-qubit mixed state *ρ*_*AB*_, concurrence and COA are known to have analytic formula^30, 40^

12 $${\mathscr{C}}({\rho }_{AB})=\,{\rm{\max }}\,\{0,{\lambda }_{1}-{\lambda }_{2}-{\lambda }_{3}-{\lambda }_{4}\},$$

13 $${{\mathscr{C}}}^{a}({\rho }_{AB})={\lambda }_{1}+{\lambda }_{2}+{\lambda }_{3}+{\lambda }_{4},$$

where *λ*_*i*_ being the eigenvalues, in decreasing order, of matrix $\sqrt{{\rho }_{AB}({\sigma }_{y}\otimes {\sigma }_{y}){\rho }_{AB}^{\ast }({\sigma }_{y}\otimes {\sigma }_{y})}$.

In ref. 40, Wootters derived an analytical formula of entanglement of formation for a two-qubit mixed state *ρ*_*AB*_

14 $${E}_{f}({\rho }_{AB})=h(\frac{1+\sqrt{1-{{\mathscr{C}}}^{2}({\rho }_{AB})}}{2}),$$

where $h(x)=-x\,\mathrm{ln}\,x-(1-x)\,\mathrm{ln}(1-x)$ is the binary entropy.

In ref. 36, Kim found ${E}_{q,s}({\rho }_{AB})$ has an analytical formula for a two-qubit mixed state, which can be expressed as a function of concurrence ${{\mathscr{C}}}_{AB}$ for *q* ≥ 1, 0 ≤ *s* ≤ 1 and *qs* ≤ 3

15 $${E}_{q,s}({\rho }_{AB})={f}_{q,s}[{\mathscr{C}}({\rho }_{AB})],$$

where the function *f*_*q*,*s*_(*x*) has the form

16 $${f}_{q,s}(x)=\frac{{[{(1+\sqrt{1-{x}^{2}})}^{q}+{(1-\sqrt{1-{x}^{2}})}^{q}]}^{s}-{2}^{qs}}{(1-q)s{2}^{qs}},$$

where 0 ≤ *x* ≤ 1.

### Main Results

In this section, we will provide our main results. First, we have following result:

Theorem 1. For any tripartite mixed state *ρ*_*ABC*_ in the Hilbert space ${ {\mathcal H} }_{{\mathscr{A}}}\otimes { {\mathcal H} }_{ {\mathcal B} }\otimes { {\mathcal H} }_{{\mathscr{C}}}$, we have

17 $${E}_{q,s}^{a}({\rho }_{A|BC})\le {E}_{q,s}^{a}({\rho }_{B|AC})+{E}_{q,s}^{a}({\rho }_{C|AB}),$$

where *q* ≥ 1 and *qs* ≥ 1.

*Proof*: Let ${\rho }_{ABC}=\,{\rm{\max }}\,{\sum }_{i}{p}_{i}{|{\psi }^{i}\rangle }_{A|BC}\langle {\psi }^{i}|$ be an optimal decomposition of ${E}_{q,s}^{a}({\rho }_{A|BC})$. That is

18 $${E}_{q,s}^{a}({\rho }_{A|BC})=\,{\rm{\max }}\sum _{i}{p}_{i}{E}_{q,s}({|{\psi }^{i}\rangle }_{A|BC}).$$

For any bipartite pure state ${|{\psi }^{i}\rangle }_{A|BC}$, the unified (*q*, *s*)-entropy entanglement ${E}_{q,s}({|{\psi }^{i}\rangle }_{A|BC})={S}_{q,s}({\rho }_{BC}^{i})$. In ref. 41, Rastegin proved that for any *q* ≥ 1 and *qs* ≥ 1, the unified (*q*, *s*)-entropy is subadditive, that is

19 $${S}_{q,s}({\rho }_{BC}^{i})\le {S}_{q,s}({\rho }_{B}^{i})+{S}_{q,s}({\rho }_{C}^{i}).$$

Combining Eq. (18) with Eq. (19), we have

20 $$\begin{array}{rcl}{E}_{q,s}^{a}({\rho }_{A|BC}) & = & \sum _{i}{p}_{i}{S}_{q,s}({\rho }_{BC}^{i})\\  & \le  & \sum _{i}{p}_{i}{S}_{q,s}({\rho }_{B}^{i})+\sum _{i}{p}_{i}{S}_{q,s}({\rho }_{C}^{i})\\  & \le  & {E}_{q,s}^{a}({\rho }_{B|AC})+{E}_{q,s}^{a}({\rho }_{C|AB}).\end{array}$$

Thus, the proof is completed.

Theorem 1. Shows a simple but interesting polygamy relation of (*q*, *s*)-EOA in a tripartite quantum system. The upper bound of (*q*, *s*)-EOA of *A*|*BC* can’t be greater than the sum of (*q*, *s*)-EOA of *B*|*AC* and (*q*, *s*)-EOA of *C*|*AB*. In particular, for a tripartite pure state ${|\psi \rangle }_{A|BC}$, the unified (*q*, *s*)-entropy entanglement ${E}_{q,s}({|\psi \rangle }_{A|BC})\le {E}_{q,s}({|\psi \rangle }_{B|AC})+{E}_{q,s}({|\psi \rangle }_{C|AB})$.

We also have the following corollary:

Corollary 1. For any mixed state ${\rho }_{{A}_{1}|{A}_{2}\cdots {A}_{n}}$ in the Hilbert space ${ {\mathcal H} }_{{A}_{1}}\otimes { {\mathcal H} }_{{A}_{2}}\otimes \cdots \otimes { {\mathcal H} }_{{A}_{n}}$, we have

21 $${E}_{q,s}^{a}({\rho }_{{A}_{1}|{A}_{2}\cdots {A}_{n}})\le \sum _{i=2}^{n}{E}_{q,s}^{a}({\rho }_{{A}_{i}|{A}_{1}\cdots {A}_{i-1}{A}_{i+1}\cdots {A}_{n}}),$$

where *q* ≥ 1 and *qs* ≥ 1.

Corollary 1. Shows a constrained relationship of (*q*, *s*)-EOA in the multi-particle system, and gives an upper bound of (*q*, *s*)-EOA of ${A}_{1}|{A}_{2}\cdots {A}_{n}$. In particular, for any pure state ${|\psi \rangle }_{{A}_{1}|{A}_{2}\cdots {A}_{n}}$, the unified (*q*, *s*)-entropy entanglement ${E}_{q,s}({|\psi \rangle }_{{A}_{1}|{A}_{2}\cdots {A}_{n}})\le {\sum }_{i=2}^{n}{E}_{q,s}({|\psi \rangle }_{{A}_{i}|{A}_{1}\cdots {A}_{i-1}{A}_{i+1}\cdots {A}_{n}})$.

Example 1: Let’s consider the general GHZ state $|GHZ\rangle =\alpha {|0\rangle }^{\otimes n}+\beta {|1\rangle }^{\otimes n}$, where |*α*|^2^ + |*β*|^2^ = 1 and *n* ≥ 3. It’s easy to show that ${\sum }_{i=2}^{n}{E}_{q,s}^{a}({\rho }_{{A}_{i}|{A}_{1}\cdots {A}_{i-1}{A}_{i+1}\cdots {A}_{n}})-{E}_{q,s}^{a}({|GHZ\rangle }_{{A}_{1}|{A}_{2}\cdots {A}_{n}})$ = $\frac{n-2}{\mathrm{(1}-q)s}[{({|\alpha |}^{2q}+{|\beta |}^{2q})}^{s}-1]\ge 0$.

Example 2: Consider a four-qubit cluster state $|{C}_{4}\rangle =\frac{1}{2}(|0000\rangle +|0011\rangle +|1100\rangle -|1111\rangle )$, which is a type of highly entangled state of four-qubit^42, 43^. The reduced states of $|{C}_{4}\rangle$ are ${\rho }_{A}={\rho }_{B}={\rho }_{C}={\rho }_{D}=\frac{I}{2}$, thus ${\sum }_{i=2}^{n}{E}_{q,s}^{a}({\rho }_{{A}_{i}|{A}_{1}\cdots {A}_{i-1}{A}_{i+1}\cdots {A}_{n}})-{E}_{q,s}^{a}(|{C}_{4}\rangle )=\frac{2}{\mathrm{(1}-q)s}[\frac{1}{(q-\mathrm{1)}s}-1]$, which is nonnegative for *q* ≥ 1 and *qs* ≥ 1.

We note that for any *n*-qubit mixed state ${\rho }_{A{C}_{1}\cdots {C}_{n}}$, the polygamy relation holds:

22 $${E}_{q,s}^{a}({\rho }_{A|{C}_{1}\cdots {C}_{n}})\le \sum _{i=1}^{n}{E}_{q,s}^{a}({\rho }_{A{C}_{i}})$$

for 1 ≤ *q* ≤ 2 and −*q* ^2^ + 4*q* − 3 ≤ *s* ≤ 1^44^. Combining Eq. (17) with Eq. (22), we have

Corollary 2. For any multi-qubit mixed state ${\rho }_{AB{C}_{1}\cdots {C}_{n}}$, the following inequality holds

23 $$\begin{array}{rcl}{E}_{q,s}^{a}({\rho }_{AB|{C}_{1}\cdots {C}_{n}}) & \le  & {E}_{q,s}^{a}({\rho }_{A|B{C}_{1}\cdots {C}_{n}})+{E}_{q,s}^{a}({\rho }_{B|A{C}_{1}\cdots {C}_{n}})\\  & \le  & 2{E}_{q,s}^{a}({\rho }_{AB})+\sum _{i=1}^{n}{E}_{q,s}^{a}({\rho }_{A{C}_{i}})+\sum _{i=1}^{n}{E}_{q,s}^{a}({\rho }_{B{C}_{i}}),\end{array}$$

where 1 ≤ *q* ≤ 2, *s* = 1.In this case, (*q*, *s*)-EOA becomes Tsallis entropy entanglement of assistance which has discussed in ref. 22.

Before our second main result, we have following lemma:

Lemma 1. For *q* = 2 and $\tfrac{1}{2}\le s\le 1$, the function *f*_*q*,*s*_(*x*) in Eq. (16) satisfies

24 $${f}_{q,s}(\sqrt{{x}^{2}+{y}^{2}})={f}_{q,s}(x)+{f}_{q,s}(y).$$

*Proof*: For *q* ≥ 2, 0 ≤ *s* ≤ 1, and *qs* ≤ 3, we have ${f}_{q,s}(\sqrt{{x}^{2}+{y}^{2}})\ge {f}_{q,s}(x)+{f}_{q,s}(y)$ ^36^. On the other hand, for 1 ≤ *q* ≤ 2 and 0 ≤ *s* ≤ 1, we have ${f}_{q,s}(\sqrt{{x}^{2}+{y}^{2}})\le {f}_{q,s}(x)+{f}_{q,s}(y)$ ^44^. The equality holds if and only if *q* = 2 and $\tfrac{1}{2}\le s\le 1$. This completes the proofs.

Next, the following result will provide a lower bound of unified (*q*, *s*)-entropy entanglement of ${|\psi \rangle }_{AB|{C}_{1}\cdots {C}_{n}}$, with respect to the bipartition between *AB* and ${C}_{1}\cdots {C}_{n}$:

Theorem 2. For any multi-qubit pure state ${|\psi \rangle }_{AB{C}_{1}\cdots {C}_{n}}$ in the Hilbert space, we have

25 $$\begin{array}{c}{E}_{q,s}({|\psi \rangle }_{AB|{C}_{1}\cdots {C}_{n}})\\ \quad \ge \,{\rm{\max }}\{\sum _{i=1}^{n}[{E}_{q,s}({\rho }_{A{C}_{i}})-{E}_{q,s}^{a}({\rho }_{B{C}_{i}})],\sum _{i=1}^{n}[{E}_{q,s}({\rho }_{B{C}_{i}})-{E}_{q,s}^{a}({\rho }_{A{C}_{i}})]\}\end{array}$$

where *q* = 2 and $$\frac{1}{2}\le s\le 1$$.

*Proof*: Given a multi-qubit pure state ${|\psi \rangle }_{AB{C}_{1}\cdots {C}_{n}}$, the unified (*q*, *s*)-entropy is subadditive for any *q* ≥ 1 and *qs* ≥ 1. Thus, the following equality holds

26 $$\begin{array}{rcl}{S}_{q,s}({\rho }_{{C}_{1}\cdots {C}_{n}}) & = & {S}_{q,s}({\rho }_{AB})\\  & \le  & {S}_{q,s}({\rho }_{A})+{S}_{q,s}({\rho }_{B})\\  & = & {S}_{q,s}({\rho }_{A})+{S}_{q,s}({\rho }_{A{C}_{1}\cdots {C}_{n}})\end{array}$$

which implies ${S}_{q,s}({\rho }_{{C}_{1}\cdots {C}_{n}})-{S}_{q,s}({\rho }_{A})\le {S}_{q,s}({\rho }_{A{C}_{1}\cdots {C}_{n}})$, and similarly, ${S}_{q,s}({\rho }_{A})-{S}_{q,s}({\rho }_{{C}_{1}\cdots {C}_{n}})\le {S}_{q,s}({\rho }_{A{C}_{1}\cdots {C}_{n}})$. Combine with the two equalities above, one obtain

27 $$|{S}_{q,s}({\rho }_{A})-{S}_{q,s}({\rho }_{{C}_{1}\cdots {C}_{n}})|\le {S}_{q,s}({\rho }_{A{C}_{1}\cdots {C}_{n}}).$$

From the definition of unified (*q*, *s*)-entropy entanglement of ${|\psi \rangle }_{AB|{C}_{1}\cdots {C}_{n}}$, with respect to the bipartition between *AB* and ${C}_{1}\cdots {C}_{n}$, we have

28 $$\begin{array}{rcl}{E}_{q,s}({|\psi \rangle }_{AB|{C}_{1}\cdots {C}_{n}}) & = & {S}_{q,s}({\rho }_{AB})\\  & \ge  & {S}_{q,s}({\rho }_{A})-{S}_{q,s}({\rho }_{B})\\  & = & {E}_{q,s}({\rho }_{A|B{C}_{1}\cdots {C}_{n}})-{E}_{q,s}({\rho }_{B|A{C}_{1}\cdots {C}_{n}}).\end{array}$$

Note that for any pure state ${|\psi \rangle }_{ABC}$ in a $2\otimes 2\otimes d$ system, the following equality holds^45, 46^

29 $${{\mathscr{C}}}^{2}({|\psi \rangle }_{ABC})={[{{\mathscr{C}}}^{a}({\rho }_{AB})]}^{2}+{{\mathscr{C}}}^{2}({\rho }_{AC}),$$

where *ρ*_*AB*_ and *ρ*_*AC*_ are the reduced matrices of state ${|\psi \rangle }_{ABC}$ respectively.

For *q* = 2 and $\frac{1}{2}\le s\le 1$, we have

30 $$\begin{array}{rcl}{E}_{q,s}({|\psi \rangle }_{AB|{C}_{1}\cdots {C}_{n}}) & = & {f}_{q,s}({\mathscr{C}}({|\psi \rangle }_{AB|{C}_{1}\cdots {C}_{n}}))\\  & = & {f}_{q,s}(\sqrt{{[{{\mathscr{C}}}^{a}({\rho }_{AB})]}^{2}+{{\mathscr{C}}}^{2}({\rho }_{AC})})\\  & = & {f}_{q,s}({{\mathscr{C}}}^{a}({\rho }_{AB}))+{f}_{q,s}({\mathscr{C}}({\rho }_{AC})),\end{array}$$

where we have used Eq. (29) in the second equality, the third equality holds is due to lemma 1. Therefore,

31 $$\begin{array}{rcl}{E}_{q,s}({|\psi \rangle }_{AB|{C}_{1}\cdots {C}_{n}}) & = & {f}_{q,s}({\mathscr{C}}({|\psi \rangle }_{AB|{C}_{1}\cdots {C}_{n}}))\\  & \le  & {f}_{q,s}(\sqrt{{[{{\mathscr{C}}}^{a}({\rho }_{AB})]}^{2}+\sum _{i=1}^{n}{{\mathscr{C}}}^{2}({\rho }_{A{C}_{i}})})\\  & \le  & {f}_{q,s}({{\mathscr{C}}}^{a}({\rho }_{AB}))+{f}_{q,s}(\sqrt{\sum _{i=1}^{n}{{\mathscr{C}}}^{2}({\rho }_{A{C}_{i}})}).\end{array}$$

Compare Eq. (30) with Eq. (31), it’s easy to see that

32 $$\begin{array}{rcl}{E}_{q,s}({|\psi \rangle }_{A|B{C}_{1}\cdots {C}_{n}})-{E}_{q,s}({|\psi \rangle }_{B|A{C}_{1}\cdots {C}_{n}}) & \ge  & {f}_{q,s}({\mathscr{C}}({\rho }_{A{C}_{1}\cdots {C}_{n}}))\\  &  & -{f}_{q,s}\{\sqrt{\sum _{i=1}^{n}{[{{\mathscr{C}}}^{a}({\rho }_{B{C}_{i}})]}^{2}}\}.\end{array}$$

We also note that

33 $$\begin{array}{rcl}{f}_{q,s}({\mathscr{C}}({\rho }_{A{C}_{1}\cdots {C}_{n}})) & \ge  & {f}_{q,s}[\sqrt{\sum _{i=1}^{n}{{\mathscr{C}}}^{2}({\rho }_{A{C}_{i}})}]\\  & = & \sum _{i=1}^{n}{f}_{q,s}({\mathscr{C}}({\rho }_{A{C}_{i}}))\\  & = & \sum _{i=1}^{n}{E}_{q,s}({\rho }_{A{C}_{i}}),\end{array}$$

where the first equality holds is due to the monogamy of concurrence^1^ and *f*_*q*,*s*_(*x*) is an increasing function for *q* ≥ 2, 0 ≤ *s* ≤ 1, and *qs* ≤ 3^36^.

On the other hand, we have

34 $$\begin{array}{rcl}{f}_{q,s}\{\sqrt{\sum _{i=1}^{n}{[{{\mathscr{C}}}^{a}({\rho }_{B{C}_{i}})]}^{2}}\} & = & \sum _{i=1}^{n}{f}_{q,s}[{{\mathscr{C}}}^{a}({\rho }_{B{C}_{i}})]\\  & \le  & \sum _{i=1}^{n}{E}_{q,s}^{a}({\rho }_{B{C}_{i}})\end{array}$$

Combine Eqs (32) and (33) with Eq. (34), we have

35 $${E}_{q,s}({|\psi \rangle }_{A|B{C}_{1}\cdots {C}_{n}})-{E}_{q,s}({|\psi \rangle }_{B|A{C}_{1}\cdots {C}_{n}})\ge \sum _{i=1}^{n}[{E}_{q,s}({\rho }_{A{C}_{i}})-{E}_{q,s}^{a}({\rho }_{B{C}_{i}})].$$

Putting Eq. (35) into Eq. (32), we obtain our result. Similarly, we have

36 $${E}_{q,s}({|\psi \rangle }_{AB|{C}_{1}\cdots {C}_{n}})\ge \sum _{i=1}^{n}[{E}_{q,s}({\rho }_{B{C}_{i}})-{E}_{q,s}^{a}({\rho }_{A{C}_{i}})]$$

Thus, the proof is completed.

Theorem 2 shows a monogamy relation for a multi-qubit pure state ${|\psi \rangle }_{AB{C}_{1}\cdots {C}_{n}}$. The lower bound of the unified (*q*, *s*)-entropy entanglement for $AB|{C}_{1}\cdots {C}_{n}$ can’t be less than the sum of the two-qubit entanglement between bipartitions of the system. In particular, if ${|\psi \rangle }_{AB{C}_{1}\cdots {C}_{n}}={|\psi \rangle }_{A{C}_{1}\cdots {C}_{n}}\otimes {|\psi \rangle }_{B}$, the entanglement of $AB|{C}_{1}\cdots {C}_{n}$ is equal to the entanglement of $A|{C}_{1}\cdots {C}_{n}$. In this case, ${E}_{q,s}({\rho }_{B{C}_{i}})=0$ for $i=1,2,\ldots ,n$. Theorem 2 becomes ${E}_{q,s}({|\psi \rangle }_{A|{C}_{1}\cdots {C}_{n}})\ge {\sum }_{i=1}^{n}{E}_{q,s}^{a}({\rho }_{A{C}_{i}})$, which is a CKW-type monogamy relation^1, 8^.

Example 3: Consider a pure state ${|\varphi \rangle }_{AB{C}_{1}{C}_{2}}=\frac{1}{\sqrt{2}}(|0000\rangle +|1001\rangle )$ in the four-qubit system. for the range *q* = 2 and $\frac{1}{2}\le s\le 1$, we have ${E}_{q,s}({\rho }_{A{C}_{1}})={E}_{q,s}^{a}({\rho }_{A{C}_{1}})=0$, and ${E}_{q,s}({\rho }_{A{C}_{2}})={E}_{q,s}^{a}({\rho }_{A{C}_{2}})=\frac{1}{s}(1-\frac{1}{{2}^{s}})$. ${E}_{q,s}({\rho }_{B{C}_{i}})={E}_{q,s}^{a}({\rho }_{B{C}_{i}})=0$ where *i* = 1, 2 and ${E}_{q,s}({|\varphi \rangle }_{AB{C}_{1}{C}_{2}})=\frac{1}{s}(1-\frac{1}{{2}^{s}})$. Therefore, we can see ${|\varphi \rangle }_{AB{C}_{1}{C}_{2}}$ saturates the inequality Eq. (25).

Example 4: Finally, let’s consider a general W state ${|W\rangle }_{{A}_{1}{A}_{2}\mathrm{..}.{A}_{n}}={a}_{1}|00\cdots 01\rangle +{a}_{2}|00\cdots 10\rangle +\cdots +{a}_{n}|10\cdots 00\rangle$ in the *n*-qubit system, where ${\sum }_{i}^{n}{|{a}_{i}|}^{2}=1$. The reduced state of subsystem *A*_1_ *A*_2_ is

37 $${\rho }_{{A}_{1}{A}_{2}}=(\begin{array}{cccc}1-{|{a}_{n-1}|}^{2}-{|{a}_{n}|}^{2} & 0 & 0 & 0\\ 0 & {|{a}_{n-1}|}^{2} & {a}_{n-1}{a}_{n}^{\ast } & 0\\ 0 & {a}_{n-1}^{\ast }{a}_{n} & {|{a}_{n}|}^{2} & 0\\ 0 & 0 & 0 & 0\end{array}),$$

which implies ${E}_{q,s}({|W\rangle }_{{A}_{1}{A}_{2}|{A}_{3}\mathrm{..}.{A}_{n}})\ge 0$. It’s also easy to show that the reduced state ${\rho }_{{A}_{i}{A}_{j}}$ is separable, where $i,j=\{1,2,\ldots ,n\}$. Thus ${E}_{q,s}({\rho }_{{A}_{1}{A}_{i}})={E}_{q,s}^{a}({\rho }_{{A}_{2}{A}_{i}})={E}_{q,s}({\rho }_{{A}_{2}{A}_{i}})={E}_{q,s}^{a}({\rho }_{{A}_{1}{A}_{i}})=0$. We find that the right side of the inequality Eq. (25) is ${\rm{\max }}\{{\sum }_{i=2}^{n}[{E}_{q,s}({\rho }_{{A}_{1}{A}_{i}})-{E}_{q,s}^{a}({\rho }_{{A}_{2}{A}_{i}})],{\sum }_{i=2}^{n}[{E}_{q,s}({\rho }_{{A}_{2}{A}_{i}})-{E}_{q,s}^{a}({\rho }_{{A}_{1}{A}_{i}})]\}$ = 0. Which mean the inequality Eq. (25) holds for the general W state.

## Conclusion

Unified (*q*, *s*)-entropy is an important generalized entropy in quantum information theory. Many entropies such as Tsallis entropy, Rényi entropy, and von Neumann entropy can be seen as a special case for unified (*q*, *s*)-entropy. In this paper, we have investigated the entanglement distribution in multi-particle systems in terms of unified (*q*, *s*)-entropy. We find that for any tripartite mixed state, the (*q*, *s*)-EOA follows a polygamy relation for *q* ≥ 1 and *qs* ≥ 1. This polygamy relation provides an upper bound for the bipartition *A*|*BC*, which also holds in multi-particle systems. Furthermore, for *q* = 2 and $\frac{1}{2}\le s\le 1$, a generalized monogamy relation is provided for unified (*q*, *s*)-entropy entanglement. This monogamy relation provides a lower bound for the bipartition $AB|{C}_{1}\cdots {C}_{n}$ in the multi-qubit system. In particular, if ${|\psi \rangle }_{AB{C}_{1}\cdots {C}_{n}}={|\psi \rangle }_{A{C}_{1}\cdots {C}_{n}}\otimes {|\psi \rangle }_{B}$, the generalized monogamy relation becomes a CKW-type monogamy relation.

Both monogamy property and polygamy property are fundamental properties of multipartite entangled states. We have studied the properties above in detail, and provided a two-parameters entropy function to study the entanglement distribution. We believe our result provides a useful methodology to understand the entanglement distribution of multi-particle entanglement.
